# Ethane-1,2-diyl bis­(pyridine-3-car­box­ylate)

**DOI:** 10.1107/S1600536809052106

**Published:** 2009-12-12

**Authors:** Iván Brito, Javier Vallejos, Matías López-Rodríguez, Alejandro Cárdenas

**Affiliations:** a Departamento de Química, Facultad de Ciencias Básicas, Universidad de Antofagasta, Casilla 170, Antofagasta - Chile; b Instituto de Bio-Orgánica ‘Antonio González’, Universidad de La Laguna, Astrofísico Francisco Sánchez N°2, La Laguna, Tenerife, Spain; c Departamento de Física, Facultad de Ciencias Básicas, Universidad de Antofagasta, Casilla 170, Antofagasta - Chile

## Abstract

The title compound, C_14_H_12_N_2_O_4_, has twofold imposed crystallographic symmetry in the solid state. The asymmetric unit contains one half-mol­ecule. An intra­molecular C—H⋯O hydrogen bond is formed between the carboxyl­ate O group and one H atom of the aromatic ring such that a five-membered ring is formed. The angle between the planes of symmetry-related aromatic rings is 44.71 (19)°.

## Related literature

For the synthesis of ditopic flexible linkers, see: Chatterjee *et al.* (2004 ▶). For the potential of coordination polymers based on this multitopic bridging ligand and metal centers as functional materials, see: Huang *et al.* (2007 ▶). For applications, see: Matsuda *et al.* (2005 ▶); Wu *et al.* (2005 ▶); Xiang *et al.* (2005 ▶). For bond-length data, see: Allen (2002 ▶).
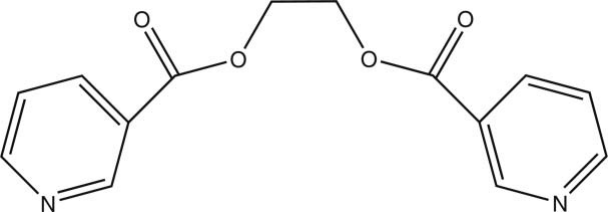


## Experimental

### 

#### Crystal data

C_14_H_12_N_2_O_4_
*M* *_r_* = 272.26
Orthorhombic, 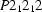

*a* = 4.0740 (14) Å
*b* = 21.3404 (7) Å
*c* = 7.395 (6) Å
*V* = 642.9 (6) Å^3^
*Z* = 2
Mo *K*α radiation
μ = 0.11 mm^−1^
*T* = 293 K
0.19 × 0.10 × 0.08 mm

#### Data collection

Nonius KappaCCD area-detector diffractometer
2298 measured reflections
874 independent reflections
694 reflections with *I* > 2σ(*I*)
*R*_int_ = 0.030

#### Refinement

*R*[*F* ^2^ > 2σ(*F* ^2^)] = 0.049
*wR*(*F* ^2^) = 0.131
*S* = 1.27
874 reflections
91 parameters
H-atom parameters constrained
Δρ_max_ = 0.16 e Å^−3^
Δρ_min_ = −0.18 e Å^−3^

### 

Data collection: *COLLECT* (Nonius, 2000 ▶); cell refinement: *DENZO-SMN* (Otwinowski & Minor, 1997 ▶); data reduction: *DENZO-SMN*; program(s) used to solve structure: *SIR97* (Altomare *et al.*, 1999 ▶); program(s) used to refine structure: *SHELXL97* (Sheldrick, 2008 ▶); molecular graphics: *ORTEP-3 for Windows* (Farrugia, 1997 ▶) and *PLATON* (Spek, 2009 ▶); software used to prepare material for publication: *WinGX* (Farrugia, 1999 ▶).

## Supplementary Material

Crystal structure: contains datablocks global, I. DOI: 10.1107/S1600536809052106/om2303sup1.cif

Structure factors: contains datablocks I. DOI: 10.1107/S1600536809052106/om2303Isup2.hkl

Additional supplementary materials:  crystallographic information; 3D view; checkCIF report

## Figures and Tables

**Table 1:** Hydrogen-bond geometry (Å, °)

*D*—H⋯*A*	*D*—H	H⋯*A*	*D*⋯*A*	*D*—H⋯*A*
C2—H2⋯O1	0.93	2.40	2.735 (5)	101

**Fig. 1.**
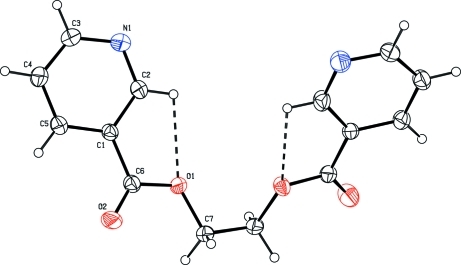
A view of the molecular structure with the atom-numbering scheme. Displacemenent ellipsoids are drawn at the 30% probability level and H atoms are shown as small spheres of arbitrary radii. Intramolecular hydrogen bonds are indicated by dotted lines. Unlabeled atoms are related to labeled atoms by the symmetry code (-x, 1 - y, z).

## supplementary crystallographic information

### Comment

In the past decade, crystalline nanoporous coordination polymers have been extensively studied for their potential applications in magnetism (Xiang, *et al.*, 2005), catalysis (Wu, *et al.*, 2005), and gas adsorption or separation (Matsuda, *et al.*, 2005). Ethanediyl pyridinecarboxylate ligands have beeen used as flexible linkers to generate metallocyclic ensembles, which showed hysteretic adsorption properties (Chatterjee *et al.*, 2004). We report here the crystal structure of the title compound which has twofold imposed crystallographic symmetry in the solid state. The asymmetric unit contains one-half of the molecule (Fig. 1). This compound crystallizes in a chiral space group, *P*2_1_2_1_2 despite the absence of a chiral center. This chirality arises from the crystal packing. A twofold rotation axis passes through the midpoint of C7 and C7(i). An intramolecular C—H···O hydrogen bond is formed between the carboxylate O group and one H-atom of the aromatic ring such that a five-membered ring is formed. The angle between the planes of symmetry-related aromatic rings is 44.71 (19). A search in the Cambridge Structural Database (version 5.30; Allen, 2002) for the title compound yielded two structures namely *catena*-(bis(µ_2_-1,2-ethanediyl bis(3-pyridinecarboxylate)-N,*N*')- bis(isothiocyanato)-cobalt(ii) trihydrate) and *catena*-(bis(µ_2_-1,2-ethanediyl bis(3-pyridinecarboxylate)-N,*N*')- bis(isothiocyanato)-cobalt(ii) tetrahydrofuran clathrate) (refcodes HEXKEB and HEXKIF, respectively) (Huang *et al.*, 2007). The most obvious differences between these coordination polymers and the uncoordinated ligand reported here are the the angles between the planes of symmetry-related aromatic rings (66.80 (12)° and 44.71 (19)°, respectively) which is a consequence of the flexible organic components of the title compound.

### Experimental

Nicotinic acid (15 g, 0.122 mol) was stirred in SOCl_2 _(40 ml) in the presence of DMF (0.6 ml) at 60 °C for 12 h. Excess thionyl chloride was removed in vacuo. Dried ethylene glycol (3.4 ml, 0.061 mol) was added. After the evolution of hydrogen chloride ended, the mixture was heated at 150 °C for 2 h. The mixture was dissolved in water, and NH_4_OH solution was added. After filtration, recrystallization in ethyl acetate gave colorless crystals. Yield 11.53 g (75%). Analysis calculated for C_14_H_12_N_2_O_4_: C: 61.76, H 4.44, N: 10.29; found: C: 61.25, H: 4.58, N: 10.15. IR (KBr, cm^-1^): (C═O) 1723 s, (C═C) 1589 m, (Ar C—C, C═N) 1424 s, (C—O) 1287 m.

### Refinement

In the absence of anomalous scatterers, 488 Friedel pairs were merged.

H atoms were positioned geometrically at distances of 0.93 (CH) and 0.97 Å (CH_2_) from the parent C atoms and refined as riding with *U*_iso_(H) = 1.2*U*_eq_(C).

50 reflections were not included in the data set as they were either partially obscured by the beam stop or were eliminated during data reduction. The material was difficult to obtain in a suitable crystalline form.

### Crystal data

**Table:**

C_14_H_12_N_2_O_4_	*F*(000) = 284
*M**_r_* = 272.26	*D*_x_ = 1.406 Mg m^−^^3^
Orthorhombic, *P*2_1_2_1_2	Mo *K*α radiation, λ = 0.71073 Å
Hall symbol: P 2 2ab	Cell parameters from 1704 reflections
*a* = 4.0740 (14) Å	θ = 1.9–27.5°
*b* = 21.3404 (7) Å	µ = 0.11 mm^−^^1^
*c* = 7.395 (6) Å	*T* = 293 K
*V* = 642.9 (6) Å^3^	Prismatic, colourless
*Z* = 2	0.19 × 0.10 × 0.08 mm

### Data collection

**Table:**

Nonius KappaCCD area-detector diffractometer	694 reflections with *I* > 2σ(*I*)
Radiation source: fine-focus sealed tube	*R*_int_ = 0.030
graphite	θ_max_ = 27.6°, θ_min_ = 1.9°
φ scans, and ω scans with κ offsets	*h* = −5→0
2298 measured reflections	*k* = −27→27
874 independent reflections	*l* = −9→9

### Refinement

**Table:**

Refinement on *F*^2^	Primary atom site location: structure-invariant direct methods
Least-squares matrix: full	Secondary atom site location: difference Fourier map
*R*[*F*^2^ > 2σ(*F*^2^)] = 0.049	Hydrogen site location: inferred from neighbouring sites
*wR*(*F*^2^) = 0.131	H-atom parameters constrained
*S* = 1.27	*w* = 1/[σ^2^(*F*_o_^2^) + (0.0408*P*)^2^ + 0.2469*P*] where *P* = (*F*_o_^2^ + 2*F*_c_^2^)/3
874 reflections	(Δ/σ)_max_ = 0.002
91 parameters	Δρ_max_ = 0.16 e Å^−^^3^
0 restraints	Δρ_min_ = −0.18 e Å^−^^3^

### Special details

**Table:**

Geometry. All s.u.'s (except the s.u. in the dihedral angle between two l.s. planes) are estimated using the full covariance matrix. The cell s.u.'s are taken into account individually in the estimation of s.u.'s in distances, angles and torsion angles; correlations between s.u.'s in cell parameters are only used when they are defined by crystal symmetry. An approximate (isotropic) treatment of cell s.u.'s is used for estimating s.u.'s involving l.s. planes.
Refinement. Refinement of *F*^2^ against ALL reflections. The weighted *R*-factor *wR* and goodness of fit *S* are based on *F*^2^, conventional *R*-factors *R* are based on *F*, with *F* set to zero for negative *F*^2^. The threshold expression of *F*^2^ > 2σ(*F*^2^) is used only for calculating *R*-factors(gt) *etc*. and is not relevant to the choice of reflections for refinement. *R*-factors based on *F*^2^ are statistically about twice as large as those based on *F*, and *R*- factors based on ALL data will be even larger.

### Fractional atomic coordinates and isotropic or equivalent isotropic displacement parameters (Å^2^)

**Table:**

	*x*	*y*	*z*	*U*_iso_*/*U*_eq_	
O1	−0.1685 (7)	0.44509 (9)	−0.0673 (3)	0.0399 (6)	
O2	−0.1110 (9)	0.34487 (11)	−0.1477 (3)	0.0619 (9)	
N1	−0.5385 (10)	0.40255 (14)	0.4274 (4)	0.0574 (10)	
C1	−0.3638 (9)	0.36912 (14)	0.1334 (4)	0.0330 (8)	
C2	−0.3991 (10)	0.41380 (16)	0.2682 (5)	0.0458 (10)	
H2	−0.3207	0.454	0.2458	0.055*	
C3	−0.6491 (10)	0.34439 (17)	0.4541 (5)	0.0497 (10)	
H3	−0.7497	0.3355	0.564	0.06*	
C4	−0.6241 (11)	0.29697 (17)	0.3305 (4)	0.0462 (10)	
H4	−0.7058	0.2573	0.3562	0.055*	
C5	−0.4756 (10)	0.30923 (15)	0.1671 (4)	0.0416 (9)	
H5	−0.451	0.2778	0.0812	0.05*	
C6	−0.2039 (9)	0.38335 (15)	−0.0413 (4)	0.0365 (8)	
C7	0.0000 (10)	0.46502 (15)	−0.2299 (4)	0.0408 (9)	
H7A	−0.1124	0.4492	−0.3362	0.049*	
H7B	0.2233	0.4493	−0.2306	0.049*	

### Atomic displacement parameters (Å^2^)

**Table:**

	*U*^11^	*U*^22^	*U*^33^	*U*^12^	*U*^13^	*U*^23^
O1	0.0527 (15)	0.0300 (12)	0.0371 (11)	−0.0025 (11)	0.0110 (13)	−0.0014 (10)
O2	0.102 (2)	0.0365 (13)	0.0474 (13)	0.0018 (16)	0.0250 (17)	−0.0097 (12)
N1	0.077 (2)	0.051 (2)	0.0441 (16)	−0.0096 (19)	0.015 (2)	−0.0089 (15)
C1	0.038 (2)	0.0291 (15)	0.0321 (15)	0.0021 (15)	−0.0016 (16)	−0.0008 (12)
C2	0.058 (3)	0.0334 (18)	0.0457 (19)	−0.0051 (19)	0.008 (2)	−0.0066 (15)
C3	0.057 (3)	0.054 (2)	0.0380 (18)	−0.009 (2)	0.008 (2)	0.0021 (17)
C4	0.055 (3)	0.0358 (18)	0.0478 (19)	−0.0023 (18)	0.003 (2)	0.0044 (15)
C5	0.049 (2)	0.0331 (17)	0.0423 (17)	−0.0009 (18)	0.0011 (19)	−0.0043 (15)
C6	0.042 (2)	0.0318 (17)	0.0360 (16)	0.0001 (16)	−0.0020 (17)	−0.0014 (14)
C7	0.047 (2)	0.0460 (18)	0.0294 (15)	−0.0102 (19)	0.0023 (18)	−0.0013 (14)

### Geometric parameters (Å, °)

**Table:**

O1—C6	1.339 (4)	C3—C4	1.368 (5)
O1—C7	1.449 (4)	C3—H3	0.93
O2—C6	1.198 (4)	C4—C5	1.376 (5)
N1—C2	1.330 (5)	C4—H4	0.93
N1—C3	1.335 (5)	C5—H5	0.93
C1—C5	1.380 (4)	C7—C7^i^	1.493 (7)
C1—C2	1.387 (4)	C7—H7A	0.97
C1—C6	1.478 (4)	C7—H7B	0.97
C2—H2	0.93		
			
C6—O1—C7	117.3 (2)	C5—C4—H4	120.7
C2—N1—C3	116.3 (3)	C4—C5—C1	118.7 (3)
C5—C1—C2	118.2 (3)	C4—C5—H5	120.6
C5—C1—C6	119.6 (3)	C1—C5—H5	120.6
C2—C1—C6	122.2 (3)	O2—C6—O1	123.1 (3)
N1—C2—C1	123.8 (3)	O2—C6—C1	124.9 (3)
N1—C2—H2	118.1	O1—C6—C1	112.0 (3)
C1—C2—H2	118.1	O1—C7—C7^i^	107.1 (3)
N1—C3—C4	124.3 (3)	O1—C7—H7A	110.3
N1—C3—H3	117.8	C7^i^—C7—H7A	110.3
C4—C3—H3	117.8	O1—C7—H7B	110.3
C3—C4—C5	118.6 (3)	C7^i^—C7—H7B	110.3
C3—C4—H4	120.7	H7A—C7—H7B	108.6
			
C3—N1—C2—C1	−0.2 (6)	C7—O1—C6—O2	−2.0 (5)
C5—C1—C2—N1	−0.9 (6)	C7—O1—C6—C1	177.0 (3)
C6—C1—C2—N1	−179.3 (4)	C5—C1—C6—O2	−13.3 (6)
C2—N1—C3—C4	0.7 (6)	C2—C1—C6—O2	165.1 (4)
N1—C3—C4—C5	0.1 (7)	C5—C1—C6—O1	167.8 (3)
C3—C4—C5—C1	−1.3 (6)	C2—C1—C6—O1	−13.9 (5)
C2—C1—C5—C4	1.7 (6)	C6—O1—C7—C7^i^	178.5 (4)
C6—C1—C5—C4	−179.9 (4)		

Symmetry codes: (i) −*x*, −*y*+1, *z*.

### Hydrogen-bond geometry (Å, °)

**Table:**

*D*—H···*A*	*D*—H	H···*A*	*D*···*A*	*D*—H···*A*
C2—H2···O1	0.93	2.40	2.735 (5)	101

## References

[bb1] Allen, F. H. (2002). *Acta Cryst.* B**58**, 380–388. 10.1107/s0108768102003890 12037359

[bb2] Altomare, A., Burla, M. C., Camalli, M., Cascarano, G. L., Giacovazzo, C., Guagliardi, A., Moliterni, A. G. G., Polidori, G. & Spagna, R. (1999). *J. Appl. Cryst.* **32**, 115–119.

[bb3] Chatterjee, B., Noveron, J. C., Resendiz, M. J. E., Liu, J., Yamamoto, T., Parker, D., Cinke, M., Nguyen, C. V., Arif, A. M. & Stang, P. J. (2004). *J. Am. Chem. Soc.* **126**, 10645–10656. 10.1021/ja0388919 15327323

[bb4] Farrugia, L. J. (1997). *J. Appl. Cryst.* **30**, 565.

[bb5] Farrugia, L. J. (1999). *J. Appl. Cryst.* **32**, 837–838.

[bb7] Huang, K., Xu, Z., Li, Y. & Zheng, H. (2007). *Cryst. Growth Des.* **7**, 2002–2004.

[bb8] Matsuda, R., Kitaura, R., Kitagawa, S., Kubota, Y., Belosludov, R. V., Kobayashi, T. C., Sakamoto, H., Chiba, T., Takata, M., Kawazoe, Y. & Mita, Y. (2005). *Nature (London)*, **436**, 238–241. 10.1038/nature03852 16015325

[bb9] Nonius (2000). *COLLECT* Nonius BV, Delft, The Netherlands.

[bb10] Otwinowski, Z. & Minor, W. (1997). *Methods in Enzymology*, Vol. 276, *Macromolecular Crystallography*, Part A, edited by C. W. Carter Jr & R. M. Sweet, pp. 307–326. New York: Academic Press.

[bb11] Sheldrick, G. M. (2008). *Acta Cryst.* A**64**, 112–122. 10.1107/S0108767307043930 18156677

[bb12] Spek, A. L. (2009). *Acta Cryst.* D**65**, 148–155. 10.1107/S090744490804362X PMC2631630 19171970

[bb13] Wu, C. D., Hu, A., Zhang, L. & Lin, W. (2005). *J. Am. Chem. Soc.* **127**, 8940–8941. 10.1021/ja052431t 15969557

[bb14] Xiang, S., Wu, X., Zhang, J., Fu, R., Hu, S. & Zhang, X. (2005). *J. Am. Chem. Soc.* **127**, 16352–16353. 10.1021/ja0546065 16305195

